# Brown Algae Phlorotannins: A Marine Alternative to Break the Oxidative Stress, Inflammation and Cancer Network

**DOI:** 10.3390/foods10071478

**Published:** 2021-06-25

**Authors:** Marcelo D. Catarino, Sónia J. Amarante, Nuno Mateus, Artur M. S. Silva, Susana M. Cardoso

**Affiliations:** 1 LAQV-REQUIMTE, Department of Chemistry, University of Aveiro, 3810-193 Aveiro, Portugal; mcatarino@ua.pt (M.D.C.); sonia.amarante@ua.pt (S.J.A.); artur.silva@ua.pt (A.M.S.S.); 2 REQUIMTE/LAQV, Department of Chemistry and Biochemistry, Faculty of Sciences, University of Porto, 4169-007 Porto, Portugal; nbmateus@fc.up.pt

**Keywords:** marine polyphenols, phlorotannins, brown seaweeds, antioxidant, anti-inflammatory, anti-tumor, oxidative stress, inflammation, cancer

## Abstract

According to the WHO, cancer was responsible for an estimated 9.6 million deaths in 2018, making it the second global leading cause of death. The main risk factors that lead to the development of this disease include poor behavioral and dietary habits, such as tobacco use, alcohol use and lack of fruit and vegetable intake, or physical inactivity. In turn, it is well known that polyphenols are deeply implicated with the lower rates of cancer in populations that consume high levels of plant derived foods. In this field, phlorotannins have been under the spotlight in recent years since they have shown exceptional bioactive properties, with great interest for application in food and pharmaceutical industries. Among their multiple bioactive properties, phlorotannins have revealed the capacity to interfere with several biochemical mechanisms that regulate oxidative stress, inflammation and tumorigenesis, which are central aspects in the pathogenesis of cancer. This versatility and ability to act either directly or indirectly at different stages and mechanisms of cancer growth make these compounds highly appealing for the development of new therapeutical strategies to address this world scourge. The present manuscript revises relevant studies focusing the effects of phlorotannins to counteract the oxidative stress–inflammation network, emphasizing their potential for application in cancer prevention and/or treatment.

## 1. Introduction

Seaweeds have been part of the human nutrition for centuries, particularly among the Far East populations such as China, Japan and Korea, to which seaweed cultivation and processing represent a great source of economic income. In contrast, Western countries have mainly explored seaweeds as a source of phycocolloids and certain fine biochemicals with industrial purposes, although the interest in their direct consumption emerged recently [1]. Marine macroalgae, from the nutritional perspective, are considered a wholesome food, since they contain important key nutrients, including carbohydrates, proteins, lipids and minerals, which are necessary for a heathy and balanced diet [2]. Another important aspect that makes seaweeds so attractive is the ease of their cultivation since they can be produced in cycles with little or no demand of fresh water nor arable land, and high growth rates that exceed those of the terrestrial crops [3]. Apart from their nutritional value, seaweeds are also a great source of many other secondary metabolites with bioactive and health promoting properties, with more than 15,000 novel compounds being reported over the last years [4].

Among them, phlorotannins represent an important group of phenolic compounds, exclusively occurring in brown algae that can form simple structures of 126 Da to very large and complex polymers. Although the biosynthetic pathway of these compounds is still not consensual, it is known that they are formed via C–C and/or C–O–C oxidative coupling of several monomeric units of phloroglucinol, which in turn is known to be biosynthesized through the acetate–malonate pathway [5]. According to the type of linkage formed between these units and the number of hydroxyl groups, phlorotannins can be classified in four sub-classes, namely phlorethols and fuhalols (ether linkages), fucols (aryl-aryl linkages), fucophlorethols (aryl-aryl and ether linkages), and eckols and carmalols (dibenzodoxine linkage) [6].

Over the recent years, phlorotannins have drawn much attention from the scientific community due to the numerous biological activities and potential industrial applications they have demonstrated, including antioxidant, anti-inflammatory, anti-obesity and several others [7]. Of these, the antitumor properties of phlorotannins are of great interest since they could represent an interesting therapeutic strategy to fight this condition that is one of the leading causes of death worldwide, second only to heart disease [8]. One of the main peculiarities of these compounds is that in addition to their direct antitumor properties, for example as pro-apoptotic, anti-proliferative, anti-metastatic or antiangiogenic effects, they can also act against tumor development indirectly due to their capacity to inhibit inflammation and oxidative stress, which are well known to play a pivotal role in the pathophysiology of cancer and several other diseases [9,10,11,12]. This versatility and capacity to interfere either directly or indirectly in multiple stages of cancer development is what makes phlorotannins so promising for the development of new pharmaceutical products and therapeutical approaches to address this world scourge.

In this context, this review presents an overview of the resourcefulness of phlorotannins to modulate numerous mechanisms underlying oxidative stress and inflammation mechanisms and highlights their contribution for preventing and tackling cancer.

## 2. Oxidative Stress, Inflammation and Cancer: How Are They Entangled?

Oxidative stress can be defined as an imbalance between the production of free radicals and reactive metabolites, commonly known as reactive oxygen and nitrogen species (ROS and RNS), and their elimination by protective mechanisms, referred to as antioxidants [13].

In a normal situation, ROS and RNS have important signaling and physiological functions in human metabolism, which is equipped with several antioxidant mechanisms to maintain their concentration in balanced levels [14]. However, when the homeostasis between antioxidants and reactive species is disturbed, overproduction of ROS and RNS becomes toxic, reacting uncontrollably with endogenous macromolecules including lipids, proteins and DNA, causing serious damage to cells and tissues [15]. These excessive levels of ROS and damages in the cellular structures lead to the activation of distinct kinases and transcription factors such as NF-κB or AP-1, which are key mediators of the pro-inflammatory signaling cascade. In turn, when activated, transcription factors promote the expression of pro-inflammatory proteins that include enzymes ((e.g., inducible nitric oxide synthase (iNOS), cyclooxygenase (COX-2) and lipoxygenase (LOX)), cytokines (e.g., tumor necrosis factor-α (TNF-α), interleukin (IL)-1β and IL-6), chemokines (e.g., monocyte chemoattractant protein 1 (MCP1), macrophage inflammatory protein 1α (MIP-1α) and IL-8), adhesion molecules (E-selectin, intercellular adhesion molecule-1 (ICAM-1) and vascular cell adhesion molecule-1 (VCAM-1)) and others, which then stimulate the surrounding cells and recruit others from the immune system [16].

The activated immune cells respond with the generation of more reactive species in an event known as oxidative burst that promote a massive release and accumulation of ROS and RNS in the damaged area and contribute to produce more pro-inflammatory mediators [17]. Until they are contained, all these events result in a self-sustaining cycle where reactive species stimulate the pro-inflammatory chemical mediators that in turn stimulate the production of more reactive species and tissue damage, establishing a perfect environment for the development of a chronic inflammation that leads to several pathological conditions and ultimately cancer.

Cancer is a multistep process defined in three main stages: initiation, promotion and progression [18]. Oxidative stress participates in all the three stages, contributing for the introduction of gene mutations and structural alterations of the DNA during the initiation stage, abnormal gene expression, blockage of cell to cell communication and modification of second messenger systems during the promotion stage, leading to an increased cell proliferation or a decreased apoptosis of the mutated cells, and finally contributing for the addition of further DNA alterations during the progression stage [19]. Moreover, it has been demonstrated that ROS are involved in the link between chronic inflammation and cancer, and that an important characteristic of tumor promoters is their ability to recruit inflammatory cells and to stimulate them to generate ROS [20].

One of the key characteristics of tumor cells is their increased survivability and capacity to resist apoptosis, which is a major barrier against tumorigenesis. Once triggered, the apoptotic signaling cascade unfolds in an orchestrated series of steps that culminate in the disruption of cellular membranes, the breakdown of nuclear and cytoplasmic skeleton, extrusion of cytosol, degradation of the chromosomes and fragmentation of the nucleus. In this process, there are essentially two types of components: the regulators, which are responsible for monitoring the extracellular and intracellular environment for conditions of normality or abnormality that influence whether a cell should live or die, and the effectors, which are responsible for the execution phase in which the cell progressively disassembles and gets consumed either by its neighbors or by phagocytic cells [21].

Many of the stimuli that trigger apoptosis converge on the mitochondria, which responds with the release of cytochrome *c*, a potent catalyst of apoptosis. These signals are however controlled by counterbalancing pro- and antiapoptotic members of the B-cell lymphoma 2 (Bcl-2) family of regulatory proteins. The antiapoptotic proteins [such as Bcl-2, B-cell lymphoma extra-large (Bcl-x_L_), and others] act by binding and suppressing the proapoptotic proteins (Bcl-2-associated X protein (Bax) and Bcl-2 homologous antagonist/killer (Bak)) in the mitochondrial outer membrane. When this suppression is lifted, Bax and Bak induce the permeabilization of the outer mitochondrial membrane causing the release of the cytochrome *c*, which in turn will activate the caspase signaling cascade, culminating in the activation of caspase-3, considered the most important of the executioner caspases, and other effector proteins that execute the selective obliteration of subcellular structures, organelles and genome [22].

In order to survive, tumor cells develop a variety of strategies that allow them to escape or limit apoptosis. One of the most common is the loss of function of tumor suppressor protein p53, which has been described as “the guardian of the genome” since it is responsible for controlling the stability of the DNA and upregulate the expression of Bax upon sensing DNA damage, thus inducing apoptosis [23]. In fact, the inactivation of this protein is commonly observed in more than 50% of human cancers and emerging evidence suggests that the dysfunction of p53 also promotes inflammation and endorses tumor immune evasion, thereby serving as an immunological driver of tumorigenesis [24,25]. In addition, tumors may evade apoptosis by upregulating the expression of antiapoptotic proteins such as Bcl-2 and Bcl-x_L_. Indeed, the overexpression of Bcl-2 has been linked to cancer resistance to chemo- and immunotherapy [26]. Other tumor evasion strategies include overexpression of survival signals, downregulation of multiple proapoptotic factors or short-circuiting the extrinsic ligand-induced death pathway. The variety of apoptosis-evading mechanisms presumably reflects the different apoptosis-inducing stimuli that tumor cell populations face and overcome during their development and evolution to the malignant state [27].

## 3. The Contribution of Phlorotannins

### 3.1. Role in Oxidative Stress and Inflammation

As phenolic compounds, the most characteristic biological property of phlorotannins is their antioxidant activity, which has been extensively explored and is encompassed by a large number of studies in literature contemplating numerous phlorotannin-rich extracts or isolated phlorotannins from multiple algal species and geographic origins, obtained with different extraction procedures and evaluated by a multitude of antioxidant assays (Table 1). Not only these compounds are capable of chelating metals and scavenging free radicals in the intracellular environment [9,28,29], but they also inhibit pro-oxidant enzymes and redox-sensitive mediators [30], and increase auto-antioxidant defenses through positive regulation of phase II detoxifying enzymes [31,32,33]. The most studied parameter for measuring the antioxidant activity is the formation of ROS. This is usually evaluated by the scavenging activities of 1,1-diphenyl-1,2-picrylhydrazyl (DPPH), 2,2′-azinobis(3-ethylbenzothiazoline-6-sulphonic acid (ABTS^+^), peroxyl (RCOO^●^), alkyl, HO^●^, and O_2_^●−^ assays and a decrease of ROS in the presence of phlorotannins has been consensually observed [28,32,34,35,36,37,38]. In many cases, their effects are even stronger when compared to well-known antioxidant compounds, such as ascorbic acid, α-tocopherol, butylated hydroxytoluene (BHT) and others. For instance, both soluble and membrane bound phlorotannin extracts from *A. nodosum* were shown to exert markedly higher DPPH^●^ scavenging activity (IC_50_ = 6.3–7.7 µg/mL) than BHT (IC_50_ = 51 µg/mL), and those from *F. vesiculosus* (IC_50_ = 3.8–4.7 µg/mL) were even stronger than ascorbic acid (IC_50_ = 6.3 µg/mL) [39]. Likewise, a phlorotannin purified extract from *F. vesiculosus*, displayed a DPPH^●^ scavenging activity better than α-tocopherol (IC_50_ = 3.76 against 5.93 µg/mL) and comparable to that of BHT (IC_50_ = 3.28 µg/mL) [40]. In a different study, the phlorotannin fraction purified from *Sargassum ringgoldianum* showed approximately five times stronger O_2_^●−^ scavenging activity than catechin (IC_50_ = 1.0 against 4.6 µg/mL) [41].

Some isolated compounds from *Ecklonia stolonifera*, namely eckstolonol, dieckol, phlorofucofuroeckol A also revealed lower IC_50_ values (8.8, 6.2 and 4.7 µM, respectively) than ascorbic acid (10.3 µM) on DPPH^●^ scavenging assay, thus evidencing remarkable antioxidant activity [38]. In turn eckol, phlorofucofuroeckol A, dieckol and 8,8′-bieckol isolated from *Eisenia bicyclis*, *Ecklonia cava* and *Ecklonia kurome*, were twice more effective scavengers of DPPH^●^ (IC_50_ = 26, 12, 13 and 15 µM, respectively) than catechin, ascorbic acid and α-tocopherol (IC_50_ = 32, 30 and 52 µM, respectively). The same tendency was observed for the O_2_^●−^ scavenging in which these compounds exhibited considerably lower IC_50_ values (10.7, 8.4, 7.6 and 6.5 µM, respectively) than resveratrol, ascorbic acid or α-tocopherol (IC_50_ = 21, 16 and 12 µM, respectively) [42].

Promising results have been observed in different cellular systems of oxidative stress as well, with several authors reporting a decrease of the intracellular ROS on cells treated either with phlorotannin-rich extracts or pure compounds [40,43,44,45,46,47,48,49]. Decrease of the DNA oxidative degradation in oxidative stress-induced cells has been described for phlorotannin extracts from species such as *F. vesiculosus*, *F. serratus* or *A. nodosum* [50,51,52,53], and pure compounds including phloroglucinol, eckol, dieckol and 2,7″-phloroglucinol-6,6′-bieckol isolated from *E. cava* [9,54,55]. Significant dose-dependent inhibitions of myeloperoxidase (MPO) activity, an enzyme that produces HOCl from H_2_O_2_ and Cl^−^, were also observed in HL-60 cells treated with phlorofucofuroeckol A, diphlororethohydroxycarmalol, 7-phloroeckol and 6,6′-bieckol prior to a pro-oxidant stimulus [34,56]. In addition to the cellular models, in vivo experiments have shown that phlorotannin extracts from *Lessonia vadosa* and *Macrocystis pyrifera* were able to prevent UV-B-induced ROS and malformations in zebrafish [57]. Likewise, compounds such as dieckol, phlorofucofuroeckol A 6,6′-bieckol, phloroglucinol, eckol, eckstolonol and triphloroethol A, all isolated from *E. cava*, were reported as effective inhibitors of intracellular ROS, lipid peroxidation and DNA damage either in ethanol, 2,2′-azobis-2-methyl-propanimidamide dihydrochloride (AAPH), high glucose or UV-B-exposed zebrafish [28,35,58,59].

In a different model, the prolonged consumption of high molecular weight phlorotannins from *Sargassum kjellmanianum* by Kunming mice (5.0 g/kg body weight/day over 30 days) was shown to significantly protect the liver against the lipid peroxidation induced either by CCl_4_ or FeSO_4_-V_C_ [60]. Notably, in a randomized controlled trial, modest improvements in the DNA damage were observed in obese people after eight weeks of consuming 400 mg/day of an *A. nodosum* phenolic-rich extract [62].

Apart from the direct antioxidant properties, the capacity of phlorotannins to boost the auto-antioxidant defenses is a subject that has also shown promising results. For instance, superoxide dismutase (SOD) and catalase (CAT), which are two important detoxifying enzymes that are usually downregulated in cells under oxidative stress, were shown to be increased on different cell lines treated either with phlorotannin-rich extracts (from *F. vesiculosus*, *F. serratus*, *Pelvetia canaliculata* and *A. nodosum*) [43,51,52] or isolated phlorotannin compounds including phloroglucinol, eckol, eckstolonol, dieckol and triphlorethol A [31,32,48,49,68,69,70]. Likewise, glutathione (GSH), GSH peroxidase (GPx), GSH reductase (GR) and GSH-S-transferase (GST), which are crucial players in the neutralization of ROS and intimately associated to the maintenance of the redox balance in living organisms, were reported in multiple studies to be upregulated in response to the treatment of *A. nodosum*, *H. elongata*, *F. serratus*, *F. vesiculosus*, *Pelvetia canaliculata*, *E. cava* and *Eisenia bicyclis* phlorotannin extracts [45,50,51,71] as well as to phloroglucinol, triphlorethol A, eckol, phlorofucofuroeckol A, 7-phloroeckol and 6,6′-bieckol [31,34,65,70,72]. As reported by the authors, it is possible that phlorotannins may interfere with the transcriptional activity of the nuclear factor erythroid 2-related factor 2 (Nrf2), which is the major regulator of the phase II detoxifying enzymes [73]. Indeed, dieckol was demonstrated to enhance the levels of detoxifying enzymes heme oxygenase-1 (HO-1), NAD(P)H:quinine oxidoreductase 1 (NQO1) and GST via upregulation of the Nrf2 transcriptional activity in H_2_O_2_-induced HepG2 cells [32]. Similar results were described for eckol, which was equally capable of stimulating the expression of HO-1 in V79-4 cells [74] and H_2_O_2_-induced HepG2 cells [31], both through activation of the nuclear translocation and transcriptional activity of Nrf2.

The phlorotannins capacity to upregulate the auto-antioxidant defenses has even been confirmed in vivo. According to Moreira et al. [63], the incorporation of *H. elongata* in restructured meat given to hypercholesterolemic rats over 35 days significantly contributed for the restoration of the levels of GR and Cu,Zn-SOD in their livers. In a different approach, the administration of 55 mg/kg/day of *L. japonica* extract to streptozotocin-induced diabetes in Sprague-Dawley rats over five days not only significantly reduced the expression of XO, but also increased the levels of GSH, GPx and GR in the liver of the treated animals [64]. Similarly, the prolonged oral administration of *Sargassum pallidum* to Wistar rats with CCl_4_-induced hepatic injury was shown to remarkably increase both GSH and SOD levels in the livers of the treated animals [61]. Upregulated SOD, GPx, GSH and CAT have also been reported in injured liver tissues of mice treated either with eckol [67] or dieckol [33,66]. Phloroglucinol was even demonstrated to ameliorate the midbrain lesions in rats with 6-hydroxydopamine-induced Parkinson’s disease, most likely through restoration of the reduced levels of CAT and GPx via stimulation of Nrf2 activity [75].

In addition to their antioxidant properties, phlorotannins are also closely related to numerous inflammatory events, being capable of inhibiting the expression of pro-inflammatory cytokines, regulate the expression and/or activity of important enzymes and even interfere with the transcriptional regulation (Table 2), making them promising therapeutic agents for mitigating inflammation and inflammatory-related diseases, which includes cancer [76,77,78].

Due to their wide distribution and ecological importance, seaweeds from the genus *Eisenia* and *Ecklonia* are perhaps the most studied in this field. The majority of the phlorotannins obtained from these seaweeds were proven of being able to inhibit the expression of cellular adhesion molecules, like ICAM-1 and VCAM-1 and cytokines, namely TNF-α, IL-1β and IL-6, in several cells of the immune system [54,79,80,81,82]. Likewise, the release of NO^●^ and PG_2_, as well as the expression of enzymes responsible for their synthesis, i.e., iNOS and COX-2, respectively, were found reduced in cells treated with *E. cava*, *E. stolonifera* and *E. arborea* extracts [83,84,85,86,87], or phlorotannin compounds from them isolated including phloroglucinol, dieckol, fucofuroeckol A, 6,6′-bieckol and phlorofucofuroeckol A [38,80,81,82,88,89]. Other seaweeds have proven their capability of inhibiting multiple pro-inflammatory markers as well. Barbosa et al. [90] recently demonstrated that phlorotannin-purified extracts from several species of *Fucus*, namely *F. vesiculosus*, *F. guiryi*, *F. spiralis* and *F. serratus*, dose-dependently inhibited the LPS-induced NO^●^ secretion in Raw 264.7 macrophages, which is in line with previous studies from Zaragozá et al. [43], who also described a dose-dependent inhibition of NO^●^ production on PMA-stimulated macrophages after being treated with a phlorotannin-rich *F. vesiculosus* ethanol extract. Likewise, phlorotannin extracts of several species of *Sargassum* including *S. horneri*, *S. sagamianum* and *S. patens* were shown to affect the expression of NO^●^, PG_2_, IL-1β, IL-6 and TNF-α in LPS-stimulated Raw 264.7 macrophages [91,92,93], whereas in HaCaT cells exposed to UV-B radiation, the addition of *S. fulvellum* ethanolic extract prevented the expression of NO^●^, PG_2_, TNF-α, COX-2 and iNOS [94]. In turn, the treatment of LPS-stimulated Raw 264.7 macrophages with different purified fractions of a polyphenol extract from *Macrocystis pyrifera* not only exerted inhibitory effects towards NO^●^, TNF-α and iNOS but also stimulated the expression of IL-10 which is an important anti-inflammatory cytokine responsible for the suppression of cytokines secretion [87].

Evidence have shown that the anti-inflammatory capacity of these compounds is much likely to be related with their modulatory effects on important transcription factors that mediate the inflammatory signaling cascade. In fact, it has been proven that the addition of *E. cava* ethanolic extract to LPS-stimulated Raw 264.7 macrophages dose-dependently diminished the levels of TNF-α, IL-1β, IL-6, iNOS, COX-2, NO^●^ and PG_2_ via inhibition of the nuclear NF-κB translocation [83]. Notably, the *E. cava* ethanolic extract ability to inhibit NF-κB nuclear translocation and binding to DNA in LPS-stimulated BV2 microglia cells was caused by the inhibition of both IκBα degradation and mitogen-activated protein kinase (MAPK) pathway, which also contributes for the regulation of pro-inflammatory cytokines biosynthesis [84]. Yayeh et al. [95] reported that the inactivation of LPS-induced NF-κB transcriptional activity in dieckol-treated Raw 264.7 macrophages occurs via inhibition of the phosphorylation of NF-κB p65 subunit and its upstream kinases namely PI3K, Akt, IKK-α/β and IκBα, which is an inhibitory route very similar to that described for phlorofucofuroeckol A in LPS-stimulated Raw 264.7 cells [96] and 6,6′-bieckol in LPS-stimulated BV2 cells [97]. In phloroglucinol-treated LPS-stimulated Raw 264.7 macrophages, the inactivation NF-κB occurred via a different path, through inhibition of the upstream kinases IKK and NF-κB inducing kinase (NIK) [54].

In a different study, Lee et al. [108] reported that in addition to the Akt/IκB-mediated NF-κB inactivation, dieckol also caused the decrease of p38 and ERK phosphorylation in three different human hepatic cell lines, which is partially in agreement with a previous work carried out on LPS-stimulated BV2 cells in which this compound only inhibited p38-MAPK but not ERK nor JNK [81]. Similar observations were described for phlorofucofuroeckol A, which inhibited the phosphorylation of p38, ERK and JNK in LPS-stimulated Raw 264.7 cells [96], and for phloroglucinol, which blocked the phosphorylation of ERK in HT1080 cells [54], both resulting in the suppression of AP-1 activity, another transcription factor involved in the regulation of some pro-inflammatory mediators such as matrix metalloproteinases, a group of enzymes involved in the tissue remodeling during chronic inflammation and cell motility further in a tumor environment. The inhibitory effects of these and other isolated phlorotannins such as eckol, eckstolonol, 6,6′-bieckol, 8,8′-bieckol, fucofuroeckol-A over NF-κB and MAPKs have been reported in multiple cell lines elicited with different pro-inflammatory stimulus, including LPS-induced BV2 microglia cells, THP-1 or Raw 264.7 macrophages, Aβ_25–35_-stimulated PC12 pheochromocytoma cells, PMA-induced MG-63 human osteosarcoma cells, high glucose-induced HUVEC endothelial cells, hypoxia-induced primary mouse hepatocytes and gamma-irradiated primary rat hepatocytes [46,65,81,82,88,89,106,109,110,111,112], suggesting that these compounds may act in a wide range of inflammatory conditions.

A common method to evaluate the anti-inflammatory properties of a potential therapeutic compound in vivo is through the induction of ear or paw edema in mice, usually with PMA or carrageenan. Indeed, using this method, Joung et al. [100] found that the topical administration of *Myagropsis myagroides* ethanolic extract in ICR mice ears (90 μg/ear) as well as 6,6′-bieckol (30 μg/ear) isolated from the same species prior to the induction of ear edema with PMA significantly suppressed the ear swelling in approximately 67% and 64%, respectively, which was very close to the effects observed for indomethacin, used as positive control (at 1 mg/ear) [100]. Similarly, attenuation of ear swelling was described for phlorotannin purified extracts of *Cystoseira sedoides*, *Cladostephus spongiosis* and *Padina pavonica* as well as pure compounds such as eckol, 6,6′-bieckol, 6,8′-bieckol, 8,8′-bieckol, phlorofucofuroeckol A and phlorofucofuroeckol B in mice ear edemas induced with several different sensitizers including arachidonic acid (AA), 12-*O*-tetradecanoylphorbol-13-acetate (TPA), oxazolone (OXA) and xylene (XYL) [98,103,104,105]. Paw edema induced by carrageenan injection was also attenuated on Wistar rats treated with intraperitoneal injection of a phlorotannin purified extract of *C. sedoides* [98]. In BALB/c mice, topical application of *Sargassum fulvellum* phlorotannin purified fraction prior to UV-B irradiation effectively suppressed the protein expression of TNF-α, COX-2 and iNOS, and their downstream products, PGE_2_ and NO^●^, respectively [94], while in UV-B-irradiated zebrafish embryos, the treatment with phloroglucinol, triphlorethol A or eckstolonol all prevented the rise of the levels of NO^●^ [107]. Likewise, diminished NO^●^ levels as well as iNOS and COX-2 expression were described on zebrafish exposed to high glucose levels and treated with dieckol [35]. Oral administration of dieckol to LPS-induced septic shock in C57BL/6 mice over seven days, was also found to significantly decrease the blood serum levels of NO^●^, PGE_2_ and high-mobility group box-1 (HMGB-1), improving their survival rates in a dose-dependent manner [101], whereas the oral administration of eckol to Kunming mice for an equal period of time effectively suppressed the expression of TNF-α, IL-1β and IL-6, and enhanced the levels of IL-10 on the CCl_4_-injured livers [67]. More recently, Sanjeewa et al. [99] reported that the expression of these pro-inflammatory cytokines as well as iNOS and COX-2 were inhibited at the mRNA level and the phosphorylation of MAPKs were suppressed in the lung tissues of particular matter and ovalbumin-exposed BALB/c mice treated with *S. horneri* ethanol extract by oral ingestion over 15 days. Notably, in an intervention study carried out on human volunteers, the oral administration of a capsule containing *A. nodosum* polyphenol extract seems to attenuate the ex vivo production of IL-6 in blood samples cultured with LPS, although no statistical significance was achieved [102].

### 3.2. Role in Cancer

The fact that phlorotannins can counteract oxidative and inflammatory states make them able to indirectly display some potential to prevent and/or mitigate oxidative stress- or inflammation-based tumor initiation and progression. Notwithstanding, the role of phlorotannins as anti-neoplastic agents has been suggested to go much further, with several authors reporting that these compounds may act as anti-proliferative, antimetastatic and anti-angiogenic agents at different types of cancer (Table 3). According to Nwosu et al. [12], both phlorotannin-purified extracts from *A. nodosum* and *Alaria esculenta* exhibited dose-dependent anti-proliferative effects on Caco-2 growth showing IC_50_ values of 33 μg/mL and 7 μg/mL, respectively. Similarly, the phenolic-rich extracts of three different *Cystoseira* species (*C. sedoides*, *C. compressa* and *C. crinita*) demonstrated promising growth inhibitory effects against three different tumor cell lines, namely A549 lung cell carcinoma cells, HCT15 colon cell carcinoma cells and MCF-7 breast adenocarcinoma cells, although their IC_50_ values (17.9–90.3 µg/mL) were much higher when compared with those of cisplatin (1.5–1.9 µg/mL), an important chemotherapeutic drug used for the treatment of several neoplasms. However, when tested on normal cells, i.e., non-tumor cells (Mardin–Darby canine kidney cells and rat fibroblasts), it was found that all the three *Cystoseira* extracts presented much lower toxicity than cisplatin, suggesting that, although their antitumor capacity may be weaker compared to the commercial drug, their specificity for tumor cells is much higher, and thus administration of higher doses could be possible for achieving the desired antitumor effect without compromising the viability of normal cells [113]. In fact, according to the work of Ahn et al. [114] carried out in xenograft mice models implanted with SKOV3 ovarian cancer cells, the oral administration of dieckol (300 mg/kg/week) was even more effective than cisplatin (9 mg/kg/week) at suppressing the tumor growth without showing any liver or kidney toxicity, while the cisplatin-treated mice revealed increased blood urea nitrogen and serum creatinine which are indicative of kidney dysfunction.

Many authors have reported a causality link between the phlorotannin content of the extracts and the extracts’ anti-proliferative effects. This is the case of the work reported by Imbs et al. [117], who found that among three different seaweeds, namely *Fucus evanescens*, *Laminaria cichorioides* and *Costaria costata*, the 60% ethanolic extract from the former, which was the highest in polyphenols (10.1% dry matter), displayed the strongest inhibitory effects on DLD-1 and HT-29 human colorectal adenocarcinoma cells growth (67% and 63%, respectively), while the extract from *L. cichorioides* harvested in May, which had the lowest polyphenol content (1.4% dry matter), exhibited the lowest inhibitory capacity (9% and 23% respectively). The authors reported identical observations for *C. costata*, since extracts from samples collected in May (phenolic content of 2.0% dry matter) exhibited slightly lower inhibitory effects than the extract from samples collected in July (phenolic content of 2.7% dry matter). These results are in agreement with a previous study carried out on HeLa cervical adenocarcinoma cells in which *Macrocystis integrifolia* and *Nereocystis leutkeana* methanolic extracts, both containing a phenolic content of 3.9 µg gallic acid equivalents/g extract, showed stronger anti-proliferative effect than the extract of *Laminaria setchellii*, which contained only 1.8 µg gallic acid equivalents/g extract [118]. More recently, it has been demonstrated that ethanol extracts from *S. muticum*, collected along the North Atlantic coast during the same harvesting season, exhibited different anti-proliferative activities towards HT-29 cells according to their total phlorotannin content, i.e., the extracts from samples collected in Norway which had the highest phlorotannin contents (approximately 5 mg/g extract) displayed growth inhibitions almost two times stronger than those observed for samples from Portugal, which contained approximately 4 mg/g extract [119].

One of the most common mechanisms responsible for phlorotannins anti-proliferative activity is their capacity to interact with multiple mediators of the apoptotic signaling cascade and thus trigger cell death by inducing apoptosis [115,116,130,131,133]. In this context, a hydrophilic fraction obtained from *F. vesiculosus* acetone extract was shown to induce apoptosis in different pancreatic cell lines through cell cycle arrest, which is comparable to the effects of common chemotherapeutic drugs clinically used, such as gemcitabine [120]. Compounds such as phloroglucinol or dieckol have been reported for their capacity to stimulate the release of cytochrome *c* from the mitochondria or the expression of several caspases including caspase-3, -7, -8 and -9 in different tumor cell lines [114,125,130,131,132,133]. Additionally, dieckol was found to dose-dependently upregulate the expression of Bid and Bim pro-apoptotic proteins in Hep3B hepatocarcinoma cells [130] and to downregulate the expression of the XIAP, FLIP and Bcl-2 anti-apoptotic proteins in SKOV3 ovarian cancer cells [114], while in MCF-7 breast cancer cells, the treatment with eckstolonol caused a dose-dependent downregulation of the anti-apoptotic protein Bcl-2 along with the upregulation of the pro-apoptotic protein Bax and tumor suppressor p53 [132]. Likewise, simultaneous increase of the pro-apoptotic proteins Bak and Bax, and decrease of the anti-apoptotic proteins Bcl-2 and Bcl-x_L_ was described in HT-29 cells treated with increasing concentrations of phloroglucinol (12.5–50 µg/mL) [133]. Yet, Kang et al. [134] reported that this compound may also exert pro-apoptotic effects on this cell line via an alternative mechanism involving the suppression of insulin-like growth factor-1 receptor (IGF-1R) expression and consequently blocking the PI3K/Akt/mTOR and Ras/ERK-MAPK signaling pathways, both playing key roles in the cell survival, growth and metabolism in colon cancer. On the other hand, phlorofucofuroeckol A seems to have the capacity to stimulate the expression of activating transcription factor 3 (ATF3), consequently inducing apoptosis in four different colorectal tumor cell lines namely HCT116, SW480, LoVo and HT-29 cells [135].

In vivo antitumor effects of eckol in a sarcoma xenograft-bearing mice model, were demonstrated to be not only resultant from its capacity to interfere with the expression of caspase-3, caspase-9, Bcl-2 and Bax, but also from its capacity to inhibit the expression of epidermal growth factor receptor (EGFR), a transmembrane protein that is usually overexpressed in many cancer types, and to stimulate the mononuclear phagocytic system, recruiting and activating dendritic cells, promoting the tumor-specific Th1 responses, increasing the CD4+/CD8+ T lymphocyte ratio, and enhancing cytotoxic T lymphocyte responses. Therefore, in addition to its anti-proliferative properties, eckol also exerts antitumor effects via stimulation of the host immune response [131]. Using a different in vivo model, Sadeeshkumar et al. [126] observed that more than activating the pro-apoptotic mechanisms (modulating the Bcl-2 family proteins, cytochrome *c* release and caspases expression), the administration of dieckol (40 mg/kg bw over 15 weeks) to Wistar rats with *N*-nitrosodiethylamine-induced hepatocarcinogenesis significantly inhibited the expression of vascular endothelial growth factor (VEGF), involved in the tumor angiogenesis, and metalloproteinases-2 and -9, both participating in the extracellular matrix processing and intimately related with tumor invasion and migration [126].

Indeed, antimetastatic effect is another characteristic of phlorotannins that greatly contributes for their antitumor properties. One of the mechanisms by which phlorotannins exert antimetastatic effects is through inhibition of the expression of matrix metalloproteinases (MMPs), which are important enzymes involved in degradation of extracellular matrix proteins and tissue remodeling and are associated with various physiological or pathological processes such as morphogenesis, angiogenesis and metastasis. Among these, MMP-2 and MMP-9 are of particular relevance since they are usually overexpressed in tumor cells and are capable of degrading type IV collagen of basement membrane, the first barrier for cancer invasion [136]. Inhibition of the expression of these two enzymes were described for *E. cava* phlorotannin-rich extracts on human fibroblasts, HT1080 fibrosarcoma cells [122] and A549 human lung cancer cells [121], while different solvent-partitioned fractions of *Ishige okamurae* were shown to not only inhibit MMP-2 and MMP-9 expressions, but also to enhance the levels of tissue inhibitor of MMPs (TIMP)-1 and TIMP-2 in HT1080 cells, at both gene and protein levels [123]. In TPA-induced human hepatoma cells, dieckol suppression of MMP-9 occurred via inhibition of the activity of ERK 1/2 and JNK kinases [128], while in HT1080 cells treated with 6,6’-bieckol both MMP-2 and MMP-9 were suppressed via inhibition of the NF-κB signaling pathway [124], evidencing the potential role of phlorotannins in inflammation-based tumor development and invasion. Identical findings were reported recently in LPS-stimulated MDA-MB-231 breast cancer cells, in which the treatment either with dieckol or phlorofucofuroeckol A (at 50 and 20 μM, respectively) significantly inhibited the cell migration and invasion via suppression of TLR-4/NF-κB-mediated expression of MMP-9 [129]. In a different breast cancer cell line (MCF-7 cells), Kim et al. [127] demonstrated that in addition to the inhibition of MMP-9 gene expression, dieckol was also capable stimulate the expression of TIMP-1 and TIMP-2, and even block the expression of VEGF, indicating that this compound may also contribute for the inhibition of tumor vascularization.

An alternative antimetastatic mechanism of dieckol was demonstrated in A549 lung cancer cells, in which migratory and invasive phenotype were remarkable abrogated via activation of tumor suppressor protein E-cadherin, an adhesion molecule that plays a key role in the epithelial-mesenchymal transition process, i.e., the mechanism by which epithelial cells lose their cell-cell adhesion characteristics and gain migratory/invasive properties [125].

## 4. Patent Review

The recovery of phlorotannins from brown macroalgae is a subject that is triggering a great economic interest, thus a domain open to technological innovations. These innovations are often translated in patents, i.e., legal documents that ensure the right of innovative inventions, in which the new product or process developed to answer a particular problem is described, with very meticulous technical information. Patent review is therefore a useful approach to have an idea of the progress made in a certain technological field. In Table 4 are described some examples of patents or patent applications filed for antioxidant, anti-inflammatory and anti-tumor products based on phlorotannins.

In 2006, Cosmax Co. (South Korea) filed the patent *Ecklonia cava* extract with high antioxidant activity describing a process for producing an extract from *E. cava* containing 10 to 15% of phlorotannins using supercritical extraction with CO_2_. The extracts produced this manner are claimed to exert high antioxidant activity and are intended for application as raw ingredients in the formulation of cosmetics and functional foods [137]. Later, in 2013, the University of Santiago de Compostela (Spain) filed the patent “Antioxidant extract from brown macroalgae and method for obtaining same” describing another method for production of phlorotannin-rich extracts. In this case, the invention relates to a method using ultrasound-assisted continuous aqueous extraction that is claimed to produce antioxidant extracts from *A. nodosum* containing 44.4 mg of phloroglucinol equivalents/g extract and *B. bifurcata* containing 62.4 mg of phloroglucinol equivalents/g extract. This invention was developed aiming the application of these extracts as active ingredients in cosmetic and food formulations [138].

Two applications for phlorotannin-based antioxidant compositions were filed by the Pukyong National University (South Korea). The first was filed in 2003 for a “Composition comprising phlorotannins isolated from the extract of *Ecklonia stolonifera* Okamura having anti-oxidative activity”. This composition contains eckstolonol, eckstolonol pentaacetate, eckol, phlorofucofuroeckol A and dieckol isolated from *E. cava* and is aimed to be used either as a food preservative, to prevent oxidative spoilage, or as a pharmaceutical ingredient for the preparation of powders, granules, tablets, capsules, suspensions, emulsions, syrups and/or other formulations with anti-aging properties via inhibition of cell membrane lipid peroxidation [139]. In 2008 they filed a second patent for “An antioxidant active composition containing compounds from *Ishige okamurae*”, describing a procedure to retrieve pure phlorotannin compounds from *I. okamurae*, namely phloroglucinol, 6,6’- bieckol and diphlorethohydroxycarmalol, through a conventional extraction method using methanol followed by solvent partitioning with different solvents and chromatography separation on Sephadex LH-20. The inventors showed that the compounds obtained with this method were highly effective at scavenging free radicals and reducing oxidative stress in different cells. As such, the inventors further describe an antioxidant composition using these compounds which is intended to be used as an active ingredient for the development of novel foods, cosmetics or pharmaceuticals targeting ROS-related diseases [140]. A third patent filed by this entity was registered in 2012, this time for an anti-inflammatory composition containing phlorotannin. This invention entitled “Anti-inflammatory composition containing phlorotannins from ecklonia stolonifera and *Ecklonia cava* extract as an effective component” consists of an anti-inflammatory composition composed of phlorofucofuroeckol-A isolated from *E. cava* and *E. stolonifera* via conventional extraction with ethanol and further purification using solvent partitioning and silica gel column chromatography. The inventors claim this composition has suppressive effects against both iNOS and COX-2 expression, addressing it as an active ingredient to be applied in the formulation of pharmaceutical products including injectables, tablets, capsules or syrups for treating inflammatory diseases. An additional application as a functional ingredient for developing health promoting beverages is also included in this document [141].

In 2007 a patent entitled “Algae extract containing polyphenols”, was filed by Diana Naturals (France), describing a method for producing an anti-inflammatory extract from *Ascophyllum* sp. and *Fucus* sp. via conventional extraction with water, following additional purifications and diafiltrations for removing alginates, iodine and low-molecular weight compounds. This extract, claimed to contain between 11 to 15% of polyphenols, was employed in an anti-inflammatory composition to be used with medicinal purposes for the prevention/treatment of inflammatory events, mainly targeting arthritis and allergy [142].

An innovative composition with anti-matrix metalloproteinase effects was registered in 2006 by Pukyong National University Industry-University Cooperation Foundation (South Korea) under the title “Composition containing phlorotannin for inhibition of matrix metalloproteinase activities”. This innovative product contains phlorotannins from the eckol type, particularly dieckol and bieckol, which are claimed to inhibit the activity of the matrix metalloproteinase-1, -2, -8 and -9 more effectively than the tetracycline type pharmacological agents. Matrix metalloproteinases are collagenolytic enzymes involved in numerous diseases, including inflammation, hyperparathyroidism, diabetes, corneal ulcer, osteoporosis, gastric ulcer, cancer and metastasis. Therefore, this composition is aimed to be applied as an active ingredient in the formulation of powders, tablets, capsules, ointment compositions, solutions, gels, pastes, patches or granules for the multiple therapeutic approaches such as treating wound, cancer metastasis, rheumatoid arthritis, inflammation, hyperparathyroidism, diabetes, corneal ulcer, osteoporosis, stomach ulcer, acne, burn, periodontal diseases, arteriosclerosis and fracture [143]. The same entity also applied for two other inventions in 2010 and 2012. The first application describes “A composition containing eckol for inhibiting a growth of cancer stem cells”, which suppresses Ras, a key intermediate in the regulation of cell proliferation and differentiation, through a Ras-Raf-ERK signal transduction system. This composition is intended to be administered alone or in combination with another anticancer drug, namely temozolomide, for preventing growth of stem cancer cells, preferentially those that have the ability to differentiate into gliobastoma or brain cancer cells [144]. The second patent is related to the previous one and describes “An anti-cancer composition containing eckol for cancer expressing k-Ras”. In this case, this composition is claimed to prevent tumorigeneses, growth and invasion of k-Ras-expressing cancers by suppressing both PI3K-Akt signaling and Ras signaling via Ras-Raf-ERK signal transduction system, without affecting other non-Ras expressing cells [145].

Overall, the research and innovation in this field of phlorotannins applied as antioxidants, anti-inflammatory or anti-tumor are slowly but steadily moving forward in a positive direction, with the Asian countries, particularly Korea, clearly leading the way since most of the patents filled are registered in this country.

## 5. Concluding Remarks

In conclusion, the data herein gathered make it clear that brown algae phlorotannins and phlorotannin-rich extracts are unanimously acknowledged by their remarkable effects on critical steps involved in the pathogenesis of cancer, either directly acting as pro-apoptotic, anti-proliferative, anti-metastatic or antiangiogenic agents or indirectly through inhibition of the multiple mechanisms of the oxidative stress-inflammation network which are intimately linked to tumorigenesis. These compounds are, therefore, endowed with a high versatility and capacity to promote and improve health status by acting on different fronts, opening a path for possible innovations on different fields. In fact, some important steps have already been taken in this direction, which can be measured by the number of phlorotannin-related patents that have been slowly but steadily emerging over the last years, meaning that these compounds are indeed object of great economic interest. This strengthens the hypothesis that phlorotannins could be used as valuable naturally occurring pharmacological alternatives for the development of novel therapeutic strategies for treating not only cancer but also other diseases.

## Figures and Tables

**Table 1:** Selected in vivo or ex vivo studies of the antioxidant activity of phlorotannins and phlorotannins extracts from brown algae.

Extract/Compound	Model	Conditions	Effect	Ref.
***Sargassum kjellmanianum***				
**EtOH 85%** **→** **Fract. CHCl_3_** **→** **PE** **→** **cellulose column** **→** **HMW phlorotannins**	CCl_4_ or FeSO_4_-V_C_-induced liver homogenate in Kumming mice	Oral administration with 5.0 g/kg before obtaining liver homogenate	↓ MDA, lipid peroxidation and swelling	[60]
***Sargassum pallidum***				
**Ac 70%** **→** **Fract. DCM** **→** **EtOAc**	Wistar rats with CCl_4_-induced hepatic injury	Oral administration of 100 mg/kg for 1 week after CCl_4_ injection	↓ MDA; ↑ GSH and SOD	[61]
***Fucus vesiculosus***				
**Ext. 1: EtOH 30%–35%** **Ext. 2: EtOH 50%–70%**	Sprague-dawley rats	Oral administration of 200 mg/kg/day for 4 weeks	↑ antioxidant defenses, PON-1 and ↓ MDA (in plasm) and ↑ SOD (in rats’ erythrocytes)	[43]
***Lessonia vadosa***				
**Ac** **→** **Ac 70%**	UVB-induced zebrafish	40 µg/mL PGE prior to irradiation (0.15 mW/cm^2^)	↓ ROS and malformations	[57]
***Macrocystis pyrifera***				
**Ac** **→** **Ac 70%**	UVB-induced zebrafish	40 µg/mL PGE prior to irradiation (0.15 mW/cm^2^)	↓ ROS and malformations	[57]
***Ascophyllum nodosum***				
**EtOH 60%** **→** **Fract. tangential flow ultrafiltration**	Human trials	Oral administration of a food-grade capsule containing 100 mg of polyphenols for 8 weeks	↓ DNA damage in obese subjects	[62]
***Himanthalia enlogata***				
**Restructured pork with 5% seaweed powder**	Hypercholesterolaemia-induced Wistar rats	Feeding ad libitum over 5 weeks	↑ CYP7A1, GR and SOD levels in liver homegenates	[63]
***Laminaria japonica***				
**Aqueous extract**	STZ-induced diabetes in Sprague-Dawley rats	Oral gavage of 100 mg/kg/d for 5 days prior to STZ injection (55 mg/kg)	↓ lipid peroxidation, XO; ↑ GSH, GPx and GR	[64]
**Isolated compounds**				
**Dieckol from *Ecklonia stolonifera***	ICR mice	Oral administration of 100 mg/kg/d for 4 days	↑ HO-1	[32]
**6,6-bieckol**	High glucose-induced zebrafish	20 µM 16 prior to glucose stimulation	↓ ROS	[35]
**Dieckol from *Ecklonia cava***	γ-radiation-induced hepatocyte cells isolated from Wistar rats	5–20 µM 1 h prior to irradiation (8 Gy/min)	↓ lipid peroxidation, DNA damage; ↑ SOD, CAT, GSH and GPx	[65]
	C57BL/KsJ-db/db mice	10 and 20 10 mg/kg/d over 14 days	↓ lipid peroxidation; ↑ SOD, GPx and CAT	[33]
	CCl_4_-induced hepatic injury in ICR mice	Oral administration of 5 mg/kg/d 6 days prior to CCl_4_ administration (0.5 mg/kg)	↓ MDA; ↑ SOD, GPx and CAT	[66]
	AAPH-induced zebrafish	50 µM 1 h prior to AAPH exposure (25 mM)	↓ ROS, lipid peroxidation and cell death	[28]
	High glucose-induced zebrafish	20 µM 16 h prior to glucose exposure (50–300 mM)	↓ ROS cell death and lipid peroxidation	[35]
	EtOH-induced zebrafish	10 and 20 µM 1 h prior to ethanol exposure (0.5%)	↓ ROS, lipid peroxidation and cell death	[58]
	UVB-induced zebrafish	50 µM 1 h prior to irradiation (50 mJ/cm)	↓ ROS and cell death	[59]
**Eckol from *Ecklonia cava***	AAPH-induced zebrafish	50 µM 1 h prior to AAPH exposure (25 mM)	↓ ROS, lipid peroxidation and cell death	[28]
	EtOH-induced zebrafish	10 and 20 µM 1 h prior to ethanol exposure (0.5%)	↓ ROS, lipid peroxidation and cell death	[58]
**Eckol commercial**	CCl_4_-induced hepatic injury in Kumming mice	Oral administration of 0.5 and 1 mg/kg/d 7 days prior to CCl_4_ injection (0.5%)	↓ MDA; ↑ SOD, GPx and GSH	[67]
**Eckstolonol from *Ecklonia cava***	AAPH-induced zebrafish	50 µM 1 h prior to AAPH exposure (25 mM)	↓ ROS, lipid peroxidation and cell death	[28]
**Phloroeckol from *Ecklonia cava***	High glucose-induced zebrafish	20 µM 16 h prior to glucose exposure (50–300 mM)	↓ ROS	[35]
**Phlorofucofuroeckol A from *Ecklonia cava***	High glucose-induced zebrafish	20 µM 16 h prior to glucose exposure (50–300 mM)	↓ ROS	[35]
	AAPH- induced zebrafish	50 µM 1 h prior to AAPH exposure (25 mM)	↓ ROS and cell death	[47]
**Phloroglucinol from *Ecklonia cava***	AAPH-induced zebrafish	50 µM 1 h prior to AAPH exposure (25 mM)	↓ ROS, lipid peroxidation and cell death	[28]
	EtOH-induced zebrafish	10 and 20 µM 1 h prior to ethanol exposure (0.5%)	↓ ROS, lipid peroxidation and cell death	[58]
**Phloroglucinol commercial**	CYP-induced in Sprague-Dawley rat	15 and 30 mg/kg/d *i.p*. over 7 + 75 mg/kg of CYP *i.p.* at day 1, 4 and 7	↓ MPO and ↑ CAT	[30]
**Triphloroethol A from *Ecklonia cava***	AAPH-induced zebrafish	50 µM 1 h prior to AAPH exposure (25 mM)	↓ ROS, lipid peroxidation and cell death	[28]

↓—decrease; ↑—increase; AAPH—2,2’-Azobis(2-amidinopropane) dihydrochloride; Ac—acetone; CAT—catalase; CCl_4_—carbon tetrachloride; CYP—cyclophosphamide; CYP7A1—cholesterol 7 alpha-hydroxylase; DCM—dichloromethane; EtOAc—ethyl acetate; EtOH—ethanol; Ext—extraction; FeSO_4_-V_C_—iron (II) sulfate-ascorbic acid; Fract.—fractionation; GPx—glutathione peroxidase; GR—glutathione reductase; GSH—glutathione; HMW—high molecular weight; HO-1—heme oxygenase-1; *i.p*.—intraperitoneal injection; MPO—myeloperoxidase; MDA—malonaldehyde; PE—petroleum ether; PON-1—paraoxonase-1; ROS—reactive oxygen species; SOD—superoxide dismutase; STZ—streptozotocin; UVB—ultraviolet-B; XO—xanthine oxidase.

**Table 2:** Selected in vivo or ex vivo studies of the anti-inflammatory activity of phlorotannins extracts and isolated phlorotannins from brown algae.

Extract/Compound	Model	Conditions	Effect	Ref.
*Cystoseira sedoides*				
EtOH 50% → Fract. PE → DCM → EtOAc	Model 1—XYL-induced mice ear edema Models 2—Carrageenan-induced paw edema in Wistar rats	Model 1—50 and 100 mg EtOAc/kg *s.c.* 30 min prior to XYL application Model 2—50 and 100 mg EtOAc/kg *i.p.* 30 min prior to carrageenan application	Model 1—↓ swelling up to 83% Model 2—↓ swelling up to 81%	[98]
*Cladostephus spongiosis*				
EtOH 50% → Fract. PE → DCM → EtOAc	Model 1—XYL-induced mice ear edema Models 2—Carrageenan-induced paw edema in Wistar rats	Model 1—50 and 100 mg EtOAc/kg *s.c.* 30 min prior to XYL Model 2—50 and 100 mg EtOAc/kg *i.p.* 30 min prior to carrageenan	Model 1—↓ swelling up to 69% Model 2—↓ swelling up to 71%	[98]
*Padina pavonica*				
EtOH 50% → Fract. PE → DCM → EtOAc	Carrageenan-induced paw edema in Wistar rats	50 and 100 mg EtOAc/kg *i.p.* 30 min prior to carrageenan	↓ swelling up to 58%	[98]
*Sargassum horneri*				
EtOH 70%	OVA + PM-induced lung inflammation in BALB/c mice	200 and 400 mg extract/kg orally administered before PM	↓ iNOS and COX-2, IL-1β, IL-6, TNF-α, pERK 1/2, p-p38-MAPK and p-JNK; ↑ HO-1 and Nrf-2	[99]
*Sargassum sagamianum*				
EtOH 80%	Ear edema in ICR mice	0–100 µg/mL of extract + 1 μg/mL LPS	↓ swelling	[92]
*Sargassum fulvellum*				
EtOH 95% → fract H_2_O → EtOAc → *n*-But	UVB-induced BALB/c mice	3 and 10 μg topically applied in the back 30 min prior to irradiation	EtOAc fraction ↓ NO^●^, PGE_2_, TNF-α, COX-2 and iNOS	[94]
*Myagropsis myagroides*				
EtOH 96%	PMA-induced ear edema in mice	90 µg topically administered 1 h prior to PMA injection	↓ swelling in 67%	[100]
*Ecklonia cava*				
EtOH 70%	Periodontitis-induced Sprague-Dawley rats	100–400 µg extract/kg/day for 8 weeks	↓ gene expression IL-1β, MMP-2 and -9 and RANKL/OPG ratio	[83]
EtOH 30% → EtOH 95%	LPS-induced septic shock in C57BL/6 mice	10–100 mg/kg/d orally administered over 7 days before *i.p.* of LPS (25 mg/kg)	↓ NO^●^, PGE_2_, iNOS, COX-2, IL-6, TNF-α, HMGB-1, and p-NIK, p-TAK1, p-IKK, p-IκB α, NF-κB-p50 and -p65; ↑ HO-1 and Nrf-2	[101]
*Ascophyllum nodosum*				
EtOH 60% → fract → food-grade capsule (101·89 mg TPC)	LPS-induced Human blood ex vivo	Oral administration of a single capsule prior to blood collection	↓ IL-6	[102]
Isolated compounds				
6,6′-Bieckol from *Eisenia arborea*	AA, TPA, OXA-induced ear edema in ICR mice	Topical application (15 nmol/µL) with each sensitizer	↓ swelling in 62%, 36% and 59%, respectively	[103]
	AA, TPA, OXA-induced ear edema	Oral gavage of 75 nmol, 18 h prior to each sensitizer	↓ swelling 42%, 34% and 18%, respectively	[104]
6,6′-bieckol from *Myagropsis myagroides*	PMA-induced ear edema in mice	30 µg topically administered, 1 h prior to PMA injection	↓ swelling in 64%	[100]
6,8′-bieckol from *Eisenia arborea*	AA, TPA, OXA-induced ear edema in ICR mice	Topical application (15 nmol/µL) with each sensitizer	↓ swelling in 56%, 66% and 78%, respectively	[103]
	AA, TPA, OXA-induced ear edema	Oral gavage of 75 nmol, 18 h prior to each sensitizer	↓ swelling 40%, 49% and 78%, respectively	[104]
8,8′-Bieckol from *Eisenia arborea*	AA, TPA, OXA-induced ear edema in ICR mice	Topical application (0.01–0.1 mg/mL) with each sensitizer	↓ swelling up to 80%, 49% and 71%, respectively	[105]
	AA, TPA, OXA-induced ear edema	Oral gavage of 75 nmol, 18 h prior to each sensitizer	↓ swelling 21%, 32% and 32%, respectively	[104]
Dieckol from *Eisenia bicyclis*	Acetic acid-induced ICR mice	20 µM, 6 h prior to *i.p.* 0.7% acetic acid	↓ CAMs and adhesion and migration of leucocytes	[79]
Dieckol from *Ecklonia cava*	γ-radiation induced hepatocytes from Wistar rats	5–20 µM 1 h prior to irradiation (8 Gy/min)	↓ NF-kB and COX-2	[65]
	CoCl_2_-induced hepatocytes from ICR mice	100 µM 30 min prior to CoCl_2_ stimulation (500 µM)	↓ COX-2 and p-p38-MAPK	[106]
	High glucose-induced zebrafish	20 µM 16 h prior to glucose	↓ NO^●^, iNOS and COX-2	[35]
	UVB-irradiated zebrafish embryos	50 µM 1 h prior to irradiation (50 mJ/cm^2^)	↓ NO^●^	[107]
Eckol comercial	CCl_4_-injured livers of Kunming mice	0.5 and 1.0 mg compound/kg/day orally administered over 7 days before *i.p.* 0.5% CCl_4_	↓ IL-1β, IL-6 and TNF-α, ↑ IL-10	[67]
Eckol from *Ecklonia cava*	UVB-irradiated zebrafish embryos	50 µM 1 h prior to irradiation (50 mJ/cm^2^)	↓ NO^●^	[107]
Eckol from *Eisenia arborea*	AA, TPA, OXA-induced ear edema in ICR mice	Topical application (0.01–0.1 mg/mL) with each sensitizer	↓ swelling up to 49%, 38% and 65%, respectively	[105]
	AA, TPA, OXA-induced ear edema	Oral gavage of 75 nmol, 18 h prior to each sensitizer	↓ swelling 13%, 40% and 19%, respectively	[104]
Eckol from *Eisenia bicyclis*	Acetic acid-induced ICR mice	20 µM, 6 h prior to *i.p.* 0.7% acetic acid	↓ CAMs and adhesion and migration of leucocytes	[79]
Eckstolonol from *Ecklonia cava*	UVB-irradiated zebrafish embryos	50 µM 1 h prior to irradiation (50 mJ/cm^2^)	↓ NO^●^	[107]
Phlorofucofuroeckol-A from *Eisenia arborea*	AA, TPA, OXA-induced ear edema in ICR mice	Topical application (0.01–0.1 mg/mL) with each sensitizer	↓ swelling up to 68%, 44% and 77%, respectively	[105]
Pholorofucofuroeckol-A from *Eisenia bicyclis*	AA, TPA, OXA-induced ear edema	Oral gavage of 75 nmol, 18 h prior to each sensitizer	↓ swelling 31%, 32% and 23%, respectively	[104]
Phlorofucofuroeckol-B from *Eisenia arborea*	AA, TPA, OXA-induced ear edema in ICR mice	Topical application (0.01–0.1 mg/mL) with each sensitizer	↓ swelling up to 78%, 56% and 72%, respectively	[105]
	AA, TPA, OXA-induced ear edema	Oral gavage of 75 nmol, 18 h prior to each sensitizer	↓ swelling 42%, 38% and 41%, respectively	[104]
Phloroglucinol commercial	Acetic acid-induced ICR mice	20 µM, 6 h prior to *i.p.* 0.7% acetic acid	↓ CAMs and adhesion and migration of leucocytes	[79]
Phloroglucinol from *Ecklonia cava*	UVB-irradiated zebrafish embryos	50 µM 1 h prior to irradiation (50 mJ/cm^2^)	↓ NO^●^	[107]
Triphlorethol A from *Ecklonia cava*	UVB-irradiated zebrafish embryos	50 µM, 1 h prior to irradiation (50 mJ/cm^2^)	↓ NO^●^	[107]

↓—decrease; ↑—increase; DCM—dichloromethane; Ac—acetone; EtOH—ethanol; EtOAc—ethyl acetate; *n*-But—*n*-buthanol; PE—petroleum ether; LPS—lipopolysaccharide; AA—arachidonic acid; TPA—12-O-tetradecanoylphorbol-13-acetate; OXA—oxazolone; OVA—ovalbumin; PMA—phorbol 12-myristate 13-acetate; HMGB1—high mobility group protein 1; CAMs—cell adhesion molecules; PGE_2_—prostaglandin E_2_; TNF-α—tumor necrosis factor α; IL-6—interleukin 6; IL-1β—interleukin 1β; IL-10—interleukin 10NO^●^—nitric oxide; COX-2—cyclooxygenase-2; iNOS—inducible nitric oxide synthase; MMP—matrix metalloproteinase; p-IKKα/β—phospho-IkB kinase; p-IkBα—phospho nuclear factor kB inhibitor α; NF-kB—nuclear factor kB; p-JNK—phospho-c-Jun N-terminal kinase; p-ERK1/2—phospho-extracellular signal—regulated kinase 1 and 2; p38-MAPK—p38 mitogen-activated protein kinases; *i.p*.—intraperitoneal injection; XYL—xylene; PM—particulate matter; *s.c.*—subcutaniously; Nrf-2—nuclear factor erythroid 2-related factor 2; HO-1—heme oxygenase; RANKL/OPG—receptor activator of NF-κB ligand-osteoprotegerin; p-NIK—phospho-NF-kappa-B-inducing kinase; p-TAK1—phospho-transforming growth factor-β-activated kinase 1; UVB—ultraviolet-B; CoCl_2_—cobalt chloride; CCl_4_—carbon tetrachloride.

**Table 3:** Antitumor activity of phlorotannins extracts and isolated phlorotannins from brown macroalgae.

Extract/Compound	Model	Conditions	Effect	Ref.
*Saccharina japonica*				
MAE with EtOH 55%	HepG2 cells	Incubation with 0.2–2 mg/mL for 24 h	↓ cell prolif.	[115]
*Ascophyllum nodosum*				
ACN:0.2% CH_2_O_2_ (1:1) → purification in SPE columns	Caco-2 cells	Incubation with 0–50 µg/mL for 72 h	↓ cell prolif. (IC_50_ = 33 μg/mL)	[12]
*Alaria esculenta*				
ACN:0.2% CH_2_O_2_ (1:1) → purification in SPE columns	Caco-2 cells	Incubation with 0–50 µg/mL fot 72 h	↓ cell prolif. (IC_50_ = 7 μg/mL)	[12]
*Cystoria sedoides*				
MAE with EtOH 50%	MCF-7 cells	Incubation with 10–200 µg/mL for 24 h	↓ cell prolif.	[116]
*Cystoseira* spp.				
H_2_O extract	Tumor cells: A549, HCT15 and MCF-7 cells Normal cells: MDCK cells and rat fibroblasts	Incubation with 25–250 µg/mL for 48 h	Tumor cells: ↓ cell prolif. (IC_50_ = 17.9–90.3 μg/mL) Normal cells: ↓ toxicity than cisplatin	[113]
*Fucus evanescens*				
EtOH 60%	DLD-1 and HT-29 cells	Incubation with 50 µg/mL for 30 d	↓ cell prolif. by 67% and 63%, respectively	[117]
*Laminaria cichorioides*				
EtOH 60%	DLD-1 and HT-29 cells	Incubation with 50 µg/mL for 30 d	↓ cell prolif. by 64% and 56%, respectively (July specimen) ↓ cell prolif. by 50% and 52%, respectively (September specimen)	[117]
*Costaria costata*				
EtOH 60%	DLD-1 and HT-29 cells	Incubation with 50 µg/mL for 30 d	↓ cell prolif. by 38% and 31%, respectively (May’s specimen) ↓ cell prolif. by 50% and 44%, respectively (July specimen)	[117]
*Macrocystis integrifolia*				
MeOH → Fract. with *n*-hex, H_2_O:EtOAc (2:3) and 1-But → subfraction of 1-But	HeLa cells	Incubation with 0.5–5 mg/mL for 72 h	↓ cell prolif. (EC_50_ = 4.11 mg/mL)	[118]
*Nereocystis leutkeana*				
MeOH → Fract. with *n*-hex → H_2_O + EtOAc (2:3) → 1-But	HeLa cells	Incubation with 0.5–5 mg/mL for 72 h	↓ cell prolif. (EC_50_ = 4.10 mg/mL)	[118]
*Laminaria setchellii*				
MeOH → Fract. with *n*-hex → H_2_O + EtOAc (2:3) → 1-But	HeLa cells	Incubation with 0.5–5 mg/mL for 72 h	↓ cell prolif. (EC_50_ = 4.53 mg/mL)	[118]
*Sargassum muticum*				
PLE with EtOH 95% → Fract. with DCM → Ac → EtOH → EtOAC	HT-29 cells	Incubation with 12.5–100 µg/mL for 24, 48 and 72 h	↓ cell prolif. by 50% after 24 h with 50 μg/mL of extract (Norway specimen)	[119]
*Fucus vesiculosus*				
Ac 99.5% → purification by HPLC	PancTu1, Panc89, Panc1 and Colo357 cells	Incubation with 0.01–100 µg/mL for 24 h	↓ cell prolif., ↑ cell cycle inhibitors (EC_50_ = 17.35, 17.5, 19.23 and 28.9 μg/mL, for each cell line, respectively)	[120]
*Ecklonia cava*				
MeOH 70% → Fract. *n*-hex → DCM → EtOAc	A549 cells	Incubation with 0–200 µg/mL for 24 or 48 h	↓ exp. of MMP-2	[121]
EtOH 95%	HDFs and HT1080 cells	Incubation with 10–1000 µg/mL for 3 d	↓ exp. of MMP-2 and -9	[122]
EtOH extract	A2780 and SKOV3 cells	Incubation with serial dilutions for 24 h	↓ cell prolif. (IC_50_ = 84.3 and 99.6 μg/mL, respectively)	[114]
*Ishige okamurae*				
EtOH → Fract. H_2_O → *n*-But → MeOH 85% → *n*-hex	HT1080 cells	Incubation with 5 or 50 µg/mL for 24 h	↓ exp. of MMP-2 and -9; ↑ exp. TIMP-1 and -2	[123]
Isolated compounds				
6,6’-Bieckol from *Ecklonia cava*	HT1080 cells	Incubation with 5–100 µg/mL for 48 h	↓ exp. of MMP-2 and -9 and exp. of NF-κB p50 and p65	[124]
Dieckol commercial	In vitro: SKOV3 cells In vivo: SKOV3-xenograft in BALB/c mice	In vitro: Incubation with 80–120 µM for 24 h In vivo: oral administration of 50 and 100 mg/mL 3 d a week for 28 days	In vitro: ↑ exp. of casp-3, -8 and -9; ↓ exp. of the XIAP, FLIP and Bcl-2 In vivo: ↓ cell prolif. and lower toxicity than cisplatin	[114]
	A549 cells	Incubation with 25–50 µg/mL for 24 h	↑ exp. of casp-3, -8 and -9, of tumor suppressor protein E-cadherin	[125]
Dieckol from *Ecklonia cava*	NDEA-induced Wistar rats	Oral administration of 40 mg/kg/d for 15 weeks	↓ exp. of Bcl-2, VEGF, MMP-2 and -9, ↑ exp. of Bax, cytochrome c and casp-3	[126]
	MCF-7 cells	Incubation with 1–100 µM for 48 h	↓ cell migration, exp. of MMP-9 and VEGF, ↑ exp. of TIMP-1 and -2	[127]
	TPA-induced SK-Hep1 cells	Incubation with 1–50 µM for 1.5 h	↓ exp. of MMP-9 and exp. of p-ERK 1/2, p-MEK 1/2 and p-JNK 1/2	[128]
	LPS-induced MDA-MB-231 cells	Incubation with 50 µM for 48 h	↓ cell invasion, exp. of TLR-4, NF-κB and MMP-2 and -9	[129]
Dieckol from *Ecklonia stolonifera*	Hep3B cells	Incubation with 70–110 µM for 24 h	↑ exp. of casp -3, -7, -8 and -9, Bid and Bim	[130]
Eckol commercial	S180-xenograft in Kumming mice	Oral administration of 0.25–1 mg/kg/d for 7 d before and 10 d after xenograft	↑ exp. of casp-3 and -9, ↓ exp. of Bcl-2, Bax, EGFR and p-EGFR	[131]
Eckstolonol from *Ecklonia cava*	MCF-7 cells	Incubation with 5–110 µM for 48 h	↓ exp. of Bcl-2, ↑ exp. of casp-3 and -9, Bax and tumor suppressor p53	[132]
Phloroglucinol commercial	HT-29 cells	Incubation with 12.5–50 µg/mL for 24 h	↑ exp. of casp-3 and -8, cytochrome c, Apaf-1, Bad and Bax, ↓ exp. of Bcl-2, Bcl-XL	[133]
	HT-29 cells	Incubation with 0–50 µg/mL for 24 h	↓ exp. of IGFR-1	[134]
Phlorofucofuroeckol A from *Eisinia bicyclis*	HCT116, SW480, LoVo and HT-29 cells	Incubation with 100 µM for 24 h	↑ exp. of ATF3	[135]
Phlorofucofuroeckol A from *Ecklonia cava*	LPS-induced MDA-MB-231 cells	Incubation with 50 µM for 48 h	↓ cell invasion, exp. of TLR-4, NF-κB and MMP-2 and -9	[129]

↓—decrease; ↑—increase; ACN—acetonitrile; Apaf-1—apoptotic protease activating factor 1; ATF3—cyclic AMP-dependent transcription factor; Bad—Bcl-2 associated agonist of cell death; Bax—Bcl-2-associated X protein; Bcl-2—B-cell lymphoma 2; Bcl-XL—B-cell lymphoma-extra-large; Bid—BH3 interacting domain death agonist; Bim—Bcl-2-like protein 11; DCM—dichloromethane; ERK 1/2 —extracellular signal—regulated kinase 1 and 2; EGFR—epiderma growth factor; CH_2_O_2_—formic acid; MeOH—methanol; Ac—acetone; EtOH—ethanol; EtOAc—ethyl acetate; *n*-hex—*n*-hexane; *n*/1-But—*n*/1-buthanol; FLIP—FLICE-inhibitory protein; HPLC—high performance liquid chromatography; IGFR-1—insulin-like growth factor 1 receptor; MAE—microwave-assisted extraction; p-MEK 1/2—phosphor-MAP kinase kinase 1 and 2; SPE—solid phase extraction; PLE—pressurized liquid extraction; NF-κB—nuclear factor κB; NDEA—N-nitrosodiethylamine; p-JNK 1/2—phospho-c-Jun N-terminal kinase 1 and 2; TLR-4—*Toll-like* receptor 4; TPA—12-O-Tetradecanoylphorbol-13-acetate; LPS—lipopolysaccharides; MMP—matrix metalloproteinase; exp.—expression; prolif.—proliferation; casp—caspase; TIMP—tissue inhibitor of metalloproteinases; VEGF—vascular endothelial growth factor; XIAP—X-linked inhibitor of apoptosis protein. Cell lines: caco-2—human colon cancer; MDCK—Mardin–Darby canine kidney; HeLa—human cervical adenocarcinoma; PancTu1—human pancreatic cancer; Colo357—human pancreatic adenosquamous carcinoma; Panc89—human pancreatic cancer; and Panc1—pancreatic carcinoma; A549—human lung adenocarcinoma; MCF-7—human breast adenocarcinoma; HCT15—human colorectal adenocarcinoma; DLD-1—human colorectal adenocarcinoma; HT-29—human colorectal adenocarcinoma; Hep3B—human hepatocellular carcinoma; HDFs—human dermal fibroblasts; SK-Hep1—human hepatocellular carcinoma; HT1080—human fibrosarcoma; HCT116—human colon cancer; SW480—human colon cancer; LoVo—human colorectal cancer; A2780—human ovarian carcinoma; SKOV3—human ovarian carcinoma; MDA-MB-231—human breast cancer; S180—murine sarcoma cancer; HepG2—human liver hepatocellular carcinoma.

**Table 4:** Patents/patent applications for antioxidant, anti-inflammatory and antitumor products containing phlorotannin extracts (PhExt) or compounds.

Product	Seaweed Species	Claims	Active Ingredients	Patent/Patent Application nr
**PhExt with antioxidant activity**	*E. cava*	Method for production of a phlorotannin extract with high antioxidant activity for cosmetic application using supercritical extraction	E. cava extract with 10%–15% phlorotannins	KR20080004758A [137]
**PhExt with antioxidant activity**	*Bifurcaria bifurcata* + *A. nodosum*	Method for production of a phlorotannin extract with antioxidant activity for cosmetic and food application using ultrasound assisted extraction	*B. bifurcate* extract containing 62.4 mg eq. of phloroglucinol/g and *A. nodosum* extract containing 44 mg eq. of phloroglucinol/g.	US2016074317A1 [138]
**Antioxidant composition**	*E. stolonifera*	Production of a composition of several phlorotannins isolated from *E. stolonifera* with high antioxidant activity for application in functional foods to prevent oxidative spoilage or pharmaceuticals for preventing aging	Eckstolonol, eckstolonol pentaacetate, eckol, phlorofucofuroeckol A and dieckol	KR20040095125A [139]
**Antioxidant composition**	*I. okamurae*	Production of an antioxidant composition containing phlorotannins isolated from *I. okamurae* for application in pharmaceuticals, food supplements and cosmetics aiming the prevention of ROS-related diseases	Phloroglucinol, 6,6’-bieckol or diphlorethohydroxycarmalol	KR20100039104A [140]
**PhExt and anti-inflammatory composition**	*F. vesiculosus* + *A. nodosum*	Method for production extracts with high content of phlorotannins and further production of an anti-inflammatory composition using these phlorotannin-rich extracts as active ingredient for application in medicaments for treating allergies and arthritis	Algal extract containing 11%–19% phlorotannins	FR2914190A1 [142]
**Anti-inflammatory composition**	*E. stolonifera* and *E. cava*	Production of a composition containing *E. stolonifera* and *E. cava* phlorotannin extracts or isolated phlorotannins for application in farmaceutical formulations aiming the inhibition of iNOS and COX-2 on inflammatory-related diseases	Phlorofucofuroeckol A	KR20120054577A [141]
**Anti-MMP composition**	Brown seaweeds	Production of a composition containing phlorotannins from brown seaweeds with inhibitory effects against matrix metalloproteinases to be used in form of powders, tablets, capsules, ointment compositions, solutions, gels, pastes, patches, granules for the treatment of wound, cancer metastasis, rheumatoid arthritis, inflammation, hyperparathyroidism, diabetes, corneal ulcer, osteoporosis, stomach ulcer, acne, burn, periodontal diseases, arteriosclerosis and fracture	Dieckol and bieckol	KR20080005711A [143]
**Anti-cancer composition**	*E. cava*	Production of a composition containing eckol isolated from *E. cava* capable of suppressing tumorigenesis, cancer growth, invasion, metastasis and malignance in k-Ras expressing cancers via inhibition of the PI3K-Akt signaling and Ras signaling through Ras-Raf-ERK signal transduction system	Eckol	KR20130104543A [145]
**Anti-cancer composition**	*E. cava*	Production of a composition containing eckol isolated from *E. cava* for prevention of cancer stem cell growth via suppression of the Ras signaling through the Ras-Raf-ERK signal transduction system	Eckol	KR20120053265A [145]

MMP—matrix metalloproteinase; PhExt—phlorotannin extrac.
